# Tubulointerstitial nephritis and uveitis in Northern Ireland

**DOI:** 10.1038/s41433-021-01677-w

**Published:** 2021-07-29

**Authors:** A. Heaney, E. McLoone, M. Williams, G. Silvestri, A. E. Courtney, D. O’Rourke, C. E. McAvoy

**Affiliations:** Belfast Health and Social Care Trust, Belfast, UK

**Keywords:** Eye manifestations, Autoimmune diseases, Predictive markers, Uveal diseases

## Abstract

**Objectives:**

This paper looks at patients with a diagnosis of tubulointerstitial nephritis and uveitis (TINU) presenting to the Northern Ireland regional adult and paediatric uveitis service in the Belfast Health and Social Care Trust. The demographic distribution, treatment required and the visual and renal outcomes of these patients are documented.

**Methods:**

Data were collected retrospectively on 24 patients with TINU using the Northern Ireland Electronic Care Record, central pathology records alongside the adult and paediatric uveitis databases from 2011 to 2021. Patients were categorised into two groups using the Mandeville classification system. Standard Uveitis Nomenclature (SUN) was used to classify the uveitis.

**Results:**

The population prevalence is at least 12.6 cases per million based on a population of 1.9 million. Nineteen of 24 cases were definite TINU and five of 24 probable. Seventeen out of 24 had biopsy-positive TIN, all of which met all of the Mandeville clinical diagnostic features required for a definite diagnosis. All but one presented with acute bilateral anterior uveitis. The paediatric cases ranged from age 12 to 18 at age of onset with a mean age of 14. Of the 18 adult onset cases, the age ranged from 20 to 76 years. The mean age of onset for the adult cases was 53 years. Of these patients 71% were female; 42% required second-line immunosuppression for ocular disease. Visual acuity was maintained. Follow-up time ranged from 3 months to 16 years. No patient developed long-term renal impairment.

**Conclusions:**

TINU is a cause of uveitis in both the paediatric and adult populations. In Northern Ireland average age with TINU was older than much of the published literature. Long-term immunosuppression for uveitis may be required as ongoing ocular, rather than renal inflammation seemed to require treatment.

## Introduction

Tubulointerstitial nephritis and uveitis (TINU) is a condition where bilateral anterior uveitis is found in patients who have tubulointerstitial nephritis (TIN). Other systemic diseases can cause ocular and renal inflammation: sarcoidosis, tuberculosis (TB), systemic lupus erythematous (SLE) and Sjogren’s. TINU is a diagnosis of exclusion. It is thought to be a relatively rare disease, accounting for <2% of all patients presenting to specialist ophthalmology uveitis clinics. The true prevalence is unknown and likely higher. Symptoms of TIN are vague and include fever, malaise, fatigue and flank pain making diagnosis difficult, and in children these symptoms may be absent.

**Fig. 1:**
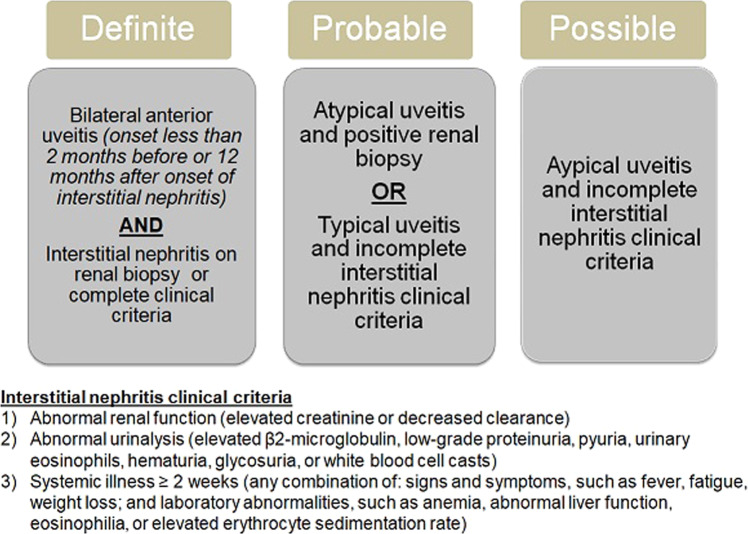
Diagnostic criteria for the classification of patients with TINU.

Mandeville, Levinson and Holland devised diagnostic criteria for the classification of patients with TINU (Fig. 1) [1].

This paper aims to provide epidemiological data on a stable population. The population in Northern Ireland is unique in that immigration is greater than emigration, therefore patients can be followed up for many years. Biopsies from all over Northern Ireland are analysed at a central pathology laboratory making data easier to collect and thoroughly analyse for the entire population.

## Methods

Analysis of the Belfast Health and Social Care Trust (BHSCT) secure paediatric ophthalmology and adult ophthalmology databases and Northern Ireland Electronic Care Record (NIECR) were used to identify all patients with a diagnosis of TINU from 2011 until the present. This was then cross-referenced with all cases of biopsy-proven TIN from the pathology department. The medical records for these patients were reviewed and data extracted for the following parameters; age at diagnosis, sex, unilateral or bilateral involvement, type of uveitis, whether a renal biopsy was carried out, creatinine levels, visual acuity at various intervals of disease and treatment provided.

Data were collected using the NIECR which allowed access to investigations carried out and clinic letters from both ophthalmology and renal teams. The available key haematological and urinalysis results were analysed. The patient’s journey from first presentation to last documented follow-up enabled analysis of disease progression over time.

Full approval was obtained from the BHSCT Standards, Quality and Audit Department. Information was managed in accordance with the BHSCT guidance on data protection.

## Results

The population prevalence of TINU in Northern Ireland is at least 12.6 cases per million based on a population of 1.9 million. Patients ranged from 12 to 76 years of age at time of diagnosis (mean 43 years).

Table 1 outlines the demographics, diagnosis and renal findings, namely creatinine at presentation, in our patient cohort. Of the 24 patients in the study, 17 (71%) were female. There appeared to be a bimodal distribution of age of onset in that six of the 24 patients presented as children and the rest presented in adulthood.

**Table 1:** Clinical features, demographics and Mandeville classification of patient with TINU.

No.	Age at diagnosis (yr)	Sex	Uveitis	Biopsy	Creatinine (normal range in μmol/L)	Mandeville classification	β2 microglobulin
1	12	F	Bilateral anterior uveitis with right papilitis	N	70 (40–68)	Probable	Elevated
2	12	F	Bilateral anterior uveitis	Y	71 (62–97)	Definite	Not tested
3	12	M	Bilateral anterior uveitis	Y	103 (39–60)	Definite	Elevated
4	13	F	Bilateral anterior uveitis	Y	299 (40–68)	Definite	Elevated
5	17	M	Bilateral anterior uveitis	Y	523 (59–104)	Definite	Not tested
6	18	M	Bilateral anterior uveitis	Y	276 (59–104)	Definite	Not tested
7	20	M	Bilateral anterior uveitis	Y	643 (59–104)	Definite	Not tested
8	36	F	Bilateral anterior uveitis	Y	1164 (45–84)	Definite	Not tested
9	37	M	Bilateral anterior uveitis	N	131 (59–104)	Definite	Elevated
10	45	M	Bilateral anterior and Intermediate uveitis	N	126 (59–104)	Probable	Not tested
11	46	F	Left anterior uveitis*	Y	116 (45–84)	Definite	Not tested
12	47	F	Bilateral anterior uveitis	Y	221 (45–84)	Definite	Not tested
13	49	F	Bilateral anterior and Intermediate uveitis	Y	439 (45–84)	Definite	Not tested
14	49	F	Bilateral anterior uveitis	N	137 (45–84)	Probable	Not tested
15	49	F	Bilateral anterior uveitis	Y	353 (45–84)	Definite	Not tested
16	53	F	Bilateral anterior uveitis - evolved to anterior and intermediate after 13 months	N	248 (45–84)	Probable	Not tested
17	53	F	Bilateral anterior uveitis	N	306 (45–84)	Definite	Not tested
18	54	F	Bilateral anterior uveitis	Y	147 (45–84)	Definite	Not tested
19	59	F	Bilateral anterior uveitis	N	286 (45–84)	Probable	Not tested
20	59	F	Bilateral anterior uveitis	Y	920 (45–84)	Definite	Not tested
21	70	F	Bilateral anterior uveitis	Y	623 (45–84)	Definite	Not tested
22	71	F	Bilateral anterior uveitis	Y	708 (45–84)	Definite	Not tested
23	74	M	Bilateral anterior uveitis	Y	433 (59–104)	Definite	Not tested
24	76	F	Bilateral anterior uveitis	Y	1136 (45–84)	Definite	Not tested

*Patient developed bilateral anterior uveitis within 1 week

All but one of our patients had symptomatic bilateral anterior uveitis on presentation to Ophthalmology; in addition, one patient also had intermediate uveitis at presentation and one other patient developed intermediate uveitis soon after initial presentation with anterior uveitis. One patient presented with left anterior uveitis 6 months after renal involvement; however, as this patient presented to the renal service first and was commenced on oral steroids this may have initially masked any inflammation in the other eye. This patient went on to develop bilateral anterior uveitis.

Seventeen of 24 patients had a renal biopsy confirming the diagnosis of TIN. All of these biopsy-positive patients also met all of the Mandeville clinical diagnostic features of TINU required for a definite diagnosis. There have been 263 renal biopsies in Northern Ireland from 2011 to present, all with a pathological diagnosis of TIN. Nineteen of 24 in this case series were definite TINU and five of 24 were classified as probable.

In Table 1, it can be seen that children have lower normal range for creatinine. Serum creatinine level is dependent on age, sex and muscle mass.

Only four of 24 patients had a β2 urinary microglobulin tested as seen in Table 1. Of these four, all showed an elevated test result. This is not part of routine practice in the adult uveitis clinic and as shown in Table 1, only one adult had this tested.

Fourteen patients (58%) presented to renal physicians first, seven (29%) presented to Ophthalmology first and three (13%) patients presented to both specialties at the same time. The patients who initially presented to the renal team developed ocular disease on average 6 months later. This delay may have occurred as patients with nephritis were treated with oral steroids initially; thus, potentially masking the uveitis. The patients who presented initially to Ophthalmology developed renal disease on average 2.5 months later. It is possible that renal disease may have been present at initial presentation but the patient was asymptomatic or had vague symptoms and renal involvement was only detected during subsequent follow-up at the tertiary uveitis service.

Visual acuity was maintained as seen in Table 2. Follow-up ranged from 3 months to 16 years, mean follow-up time was 4.5 years. One patient had cystoid macular oedema as a complication of anterior uveitis. No patients had glaucoma. One 71-year-old patient had age-related macular degenerative changes with subretinal fluid.

**Table 2:** Visual outcomes, follow-up and treatment of TINU patients.

	Snellen VA at presentation		Snellen VA at 1 year		Number of	Snellen VA at last follow-up		Current treatment
	Right	Left	Right	Left	years of follow-up	Right	Left	
1	6/9	6/6	6/6	6/6	4	6/6 − 1	6/7.5	Discharged
2	6/9	6/9	6/12	6/12	2	6/12	6/12	Predforte and oral Prednisolone 1 mg OD
3	6/18	6/9	6/18	6/12	5	6/12	6/15	Maxidex OD to both eyes. Awaiting left cataract surgery
4	6/6	6/5	6/6	6/6	16	6/7.5	6/6	Mycophenolate 1.5 g BD and Timolol 0.5% BD Right eye
5	6/6	6/6	6/6	6/5	4	6/7.5	6/5	Discharged
6	6/6	6/6	6/9 − 2	6/6	7	6/7.5	6/7.5	Mycophenolate 1.5 g BD, Prednisolone 5 mg OD, Humira alt weeks, Pred-forte, Mydrilate
7	6/5	6/5	6/6 + 4	6/5 − 3	9	6/5	6/5	Mycophenolate 1 gm BD
8	6/24	6/24	6/6 − 2	6/9 − 2	6	6/4.8	6/9.5	Methotrexate 15 mg weekly, Prednisolone 5 mg. Subtenon triamcinolone injection.
9	6/7.5	6/6	6/6 + 2	6/5	3	6/5	6/5	Sub Cut Humira 40 mg every 2 weeks
10	Not known		6/5	6/4	10	6/7.5	6/5	Discharged
11	6/6	6/5	6/6	6/4.8	3	6/6	6/5	Discharged
12	6/24	6/6	<1 Year since presentation		1	6/6	6/5	Discharged
13	6/6 + 2	6/6 + 3	6/9 + 1	6/9 − 1	8	6/9.5	6/5	Predsol 0.5% bd to both eyes
14	6/6	6/6	6/6 + 2	6/9 + 1	8	6/7.5	6/7.5	Mycophenolate. Prednisolone 5 mg OD. Orbital floor Kenalog injection.
15	6/4.8	6/4.8	<1 Year since presentation		0.25	6/4.8	6/4.8	Mycophenolate for eyes
16	6/5	6/5	<1 Year since presentation		0.33	6/4.8	6/4.8	Mycophenolate for eyes
17	6/7.5	6/7.5	6/7.5	6/7.5	1	6/19	6/7.5	Oral Prednisolone
18	6/6	6/5	6/5	6/5	10	6/12	6/9.5	Predforte twice a day and Ganfort once a day.
19	6/9	6/9	6/6	6/6	4	6/9	6/6	Discharged
20	6/6	6/6	6/6	6/6	3	6/5	6/5	Discharged
21	6/7.5	6/6	6/6	6/6	2	6/6	6/6	Lost to follow-up
22	6/6	6/6	6/6	6/6	1	6/4.8	6/6	Discharged
23	6/6	6/6	<1 Year since presentation		0.33	6/6	6/6	Discharged
24	6/7.6	6/7.6	<1 Year since presentation		0.33	6/7.6	6/7.6	Discharged

Of the 24 patients studied, 10 required second-line immunosuppression, three required long-term topical treatment and 11 required no long-term treatment as seen in Table 2.

## Discussion

The patient cohort is population-based as the Belfast Adult and Paediatric Uveitis Regional Services captures patients from across Northern Ireland. According to migration statistics, immigration in Northern Ireland (NI) is greater than emigration making our population ideal for epidemiological studies. The prevalence of TINU in this population is at least 12.6 cases per million based on Northern Ireland Statistics and Research Agency (NISRA) population estimate of 1.9 million, over a 10-year period from 2011 to 2021. This may be an underestimation for reasons discussed below. Previous publications on TINU have not been population based.

In 2008 Sinnamon et al. reported a prevalence of 3.5 cases per million population in Northern Ireland [2]. The increase in TINU in this cohort, when compared with the population in Sinnamon et al., may be because the pathology records were not easily cross-referenced with ophthalmology findings as this paper was written before the use of the Northern Ireland Electronic Care Record (NIECR) database. This system allows easier pick up of abnormal urea and electrolyte results in patients who present to their GP with non-specific symptoms and subsequently develop uveitis.

TINU is likely under-recognised making it highly likely that any incidence and prevalence figures are likely underestimates [3]. Okafor et al. state an estimated prevalence of TINU ranging from <0.1% to 2% in ‘all age’ populations and up to 2.3% in paediatric populations within patients attending specialist uveitis services. Limited data result in inaccurate estimates [4].

In the study, 71% of the patients were female and 29% were male. This female predominance has also been reported by other authors [1, 5].

The cohort of TINU patients in Northern Ireland differs from the published literature in that the average age of our patients was 43 years. The reasons for this are unclear and may include genetic and environmental factors. Mandeville et al. and Mackenson et al. reported a median age of 15 years in their TINU cohorts [1, 3]. Our population cohort is similar in age to a previously reported Northern Ireland cohort by Sinnamon et al. in 2008 [2]. This paper looked at cases between 1988 and 2006 which may indicate that within Northern Ireland, with a central pathology database and a stable population, that TINU is actually more prevalent in an older population than described in the literature.

The case series presented in this paper appears to have a bimodal distribution of age of onset. The decades with highest incidence are the second and fourth. Of the 24 patients in this study, six presented as children and 18 patients presented in adulthood. The paediatric cases ranged from age 12 to 18 at age of onset, three of these were female and three male. Five met the gold standard for having a renal biopsy, which in all five cases was positive for TIN; they also presented with typical bilateral anterior uveitis. One of the paediatric cases did not have a biopsy, however, did present with bilateral anterior uveitis with right papilitis and was classified using the Mandeville criteria as having probable TINU as they had incomplete clinical criteria. Of the 18 adult onset cases, the age ranged from 20 to 76 years. The mean age of onset for this group was 53 years. There were four males and 14 females in this cohort. Six of these patients did not have the gold standard renal biopsy and 12 had a positive renal biopsy for TIN. All of the biopsy-positive patients also presented with typical bilateral anterior uveitis. All of the biopsy-positive patients fulfilled the Mandeville criteria for clinical TINU. Of the patients who did not have a biopsy, two were classified as definite TINU as despite not having a biopsy, they presented with typical bilateral anterior uveitis and complete clinical criteria as per the Mandeville criteria. The other four were defined as probable TINU as they had bilateral anterior uveitis but incomplete clinical criteria.

Patients were categorised into two groups using the Mandeville criteria. Although this is un-validated, it represents an attempt to encourage precision in the recognition of TINU as suggested by Clive and Vanguri [6]. Histological evidence of acute TIN should be sought for a definitive diagnosis, however, an invasive procedure such as renal biopsy may not be suitable for all patients and this should be judged on an individual basis. In selected cases, the risks of a renal biopsy may outweigh the benefits and in such instance the renal physicians make a clinical diagnosis based on the patient’s renal function, urinalysis and the absence of other causes to make a clinical diagnosis of TIN. Typical biopsy findings include interstitial oedema with active interstitial inflammation mainly composed of plasma cells, lymphocytes and eosinophils [2]. The Mandeville criteria is widely used as Mandeville et al. have been the only group to publish evaluation criteria for TINU syndrome to date [3, 5–7].

Seventeen of the 24 patients in this study had the gold standard biopsy which was positive for TIN in all 17 cases (Table 1). These patients also presented with bilateral anterior uveitis and therefore according to the Mandeville classification would be defined as having definite TINU (Fig. 1). Interestingly, these patients had complete clinical criteria as per the Mandeville criteria. The other seven patients did not have a biopsy as it was deemed unnecessary by the renal team and so were classified using the un-validated Mandeville criteria; two were classed as definite because they had typical anterior uveitis and complete clinical criteria and five probable as they had typical anterior uveitis and incomplete clinical criteria.

The clinical criteria can also be met by other conditions making it difficult to fully validate the use of the current criteria for the diagnosis of TINU. It is possible that a patient may present to ophthalmology with bilateral anterior uveitis and coincidentally have a UTI; this would mean they could meet the clinical criteria for TINU despite this not being the correct diagnosis. In this cohort, 17 of 24 patients did have a biopsy confirming TIN reaching the gold standard. TINU is a diagnosis of exclusion which highlights the importance of systemic investigation. Two patients were excluded from this data set as one had Sjogren’s and another had a diagnosis of sarcoidosis. Every patient presenting to the regional adult uveitis service has a detailed questionnaire completed which aims to identify the potential for any other underlying systemic conditions. Selected patients then have the following bloods sent; urea and electrolytes, full blood count, liver profile, bone profile, C-reactive protein, ESR, Angiotensin converting enzyme and syphilis serology. If renal involvement is suspected, the patients also get a vasculitis screen and antinuclear antibodies tested for conditions like Sjogren’s. Patients also have urinalysis carried out and children get a urine sample sent for β2 microglobulin, this is not common practice in adults as seen in Table 1.

There are currently no non-invasive tests of high diagnostic value in TINU. The kidneys display an uptake on gallium scintigraphy, however, the specificity is weak. Increased urinary *N*-acetylglucosaminidase (NAG) and β2 microglobulin also lack sufficient specificity to obviate the need for kidney biopsy [7]. The major prerequisite for making the diagnosis is the presence of both TIN and uveitis, in the absence of other systemic disorders with which they are known to be associated.

Mandeville et al. reported that ocular symptoms preceded systemic symptoms in 21% of cases, with the systemic symptoms developing up to 14 months later in as many as 65% of cases [1].

In line with published literature, patients tended to present with acute onset bilateral anterior uveitis. Two patients were also noted to have an intermediate uveitis. Fourteen patients presented first to renal physicians following the discovery of renal impairment as part of investigation of typically vague symptoms such as fatigue. These patients over time developed symptoms of uveitis and were subsequently sent to Ophthalmology clinic for review. On average, they presented to Ophthalmology 6 months later. This delay is likely due to the initial oral steroids for renal impairment masking symptoms of uveitis with patients only presenting with eye symptoms following the reduction or cessation of steroids. This suggests that the Mandeville criteria can be useful in the absence of a renal biopsy. Not all patients will undergo renal biopsy and may be treated empirically with steroids to see if renal function improves. It may be useful if such patients are screened for uveitis in the eye clinic. There is currently no formal pathway for this other than the renal physicians requesting ophthalmologists input, usually only if the patient has ocular symptoms. Seven patients presented to eye casualty initially with symptoms of uveitis and when followed up at the specialist uveitis clinic, blood tests indicated co-existing acute kidney injury prompting referral to nephrology for further investigation. Three patients presented to both specialties at the same time. This highlights the need for nephrologists to be aware of the potential significance of ocular symptoms in the setting of nephritis. The detection of asymptomatic uveitis may however help the renal physician diagnose TINU and this has now been highlighted and a new pathway developed so that renal physicians can ask for prompt ophthalmological input in patients with suspected TIN and no ocular symptoms prior to the initiation of oral steroids.

All children presenting to the regional paediatric uveitis clinic with bilateral anterior uveitis routinely have creatinine and urinary β2 microglobulin checked to assess for possible TINU. Children also have a comprehensive questionnaire and work up completed to screen for other conditions such as HLA B27-positive and juvenile idiopathic arthritis-associated uveitis. While β2 microglobulin is not specific for TINU, it is often raised as there is tubular disease. Elevated urinary β2 microglobulin is a marker for interstitial nephritis and therefore may be helpful in the diagnosis of TINU syndrome, particularly when a renal biopsy is not indicated [8]. TIN leads to excretion of β2 microglobulin without resorption resulting in an elevated level in urine. It may remain elevated for months after the routine urinalysis and serum creatinine have returned to normal [1]. However, sensitivity and specificity of urinary β2 microglobulin measurement for this diagnosis are not known and it is not routinely used in the adult population in Northern Ireland [3]. Hettinga et al. concluded that measuring serum creatinine and β2 microglobulin is a sensitive screening tool for the diagnosis of definitive and/or probable TINU syndrome in young patients with uveitis and combining these two measurements increases the positive predictive value [9].

It is reassuring to note that best-corrected visual acuity was maintained at 6/12 or better in all affected eyes in our cohort except for one 76-year-old patient who had co-existing age-related macular degeneration. There were no cases of choroiditis. In this cohort, 42% of patients required second-line immunosuppression for their ocular disease.

One of the strengths of this paper is that most of our patients were followed up over a long period of time, follow-up time ranging from 3 months to 16 years with a median follow-up of 3.5 years. The patients who required immunosuppression were treated with Mycophenolate, Methotrexate or Adalimumab with or without oral Prednisolone for chronic ocular inflammation which had proven difficult to control and which flared on attempted reduction of immunosuppressive therapy. These data suggest that patients with TINU can develop ongoing chronic ocular inflammation long after renal disease has recovered, highlighting the need for long-term follow-up of TINU patients [10, 11].

Recurrence of uveitis has been reported to occur in up to 56% of patients with TINU [1, 12]. Most recurrences occur within the first few months of stopping therapy. Long-term ocular complications are fortunately rare. Renal outcomes are generally good with nephritis often spontaneously resolving, but there have been cases of chronic renal failure following TINU [1, 12]. In contrast to uveitis, nephritis rarely recurs. None of the patients in this study required dialysis or long-term renal follow-up.

One of the limitations of our study is that it is retrospective with a variable follow-up time from 3 months to 16 years. However, this study is the first report capturing both adults and children with TINU in a defined population, enabling us to estimate population prevalence for this rare disease. In addition, as patients in Northern Ireland tend to remain with the same ophthalmology service for many years, it has enabled us to report on long-term visual outcomes and treatment over a median follow-up period of 3.5 years making this paper unique by providing data on a population level.

In conclusion, TINU is a rare and likely to be under-diagnosed disease, which affects females more than males and can occur in an older population than previously reported. Patients tend to recover well from the nephritis; however, there is a high risk of long-term ocular inflammation, in some cases requiring second-line immunosuppression. Reassuringly, vision seems to be maintained in the long term. As patients may present to ophthalmic services initially, it is important to consider, in the correct clinical context, creatinine and urine dipstick testing in all patients presenting with bilateral anterior uveitis, irrespective of age, so as not to miss co-existing renal disease. Close collaboration between renal physicians and ophthalmologists is essential in both the diagnosis and management of TINU.

### Summary

#### What was known before

- TINU likely underdiagnosed female-predominance. Renal disease often resolved with no long-term renal complications. Use of urinary β2 microglobulin can be helpful in diagnosis.

#### What this study adds

- Older age of diagnosis - TINU must also be considered in older patients. Study of adults and children allowing a population prevalence - few papers have a unique population like Northern Ireland where we have data on all adult and paediatric TINU patients. Long follow-up time - many patients required long-term immunosuppression due to ocular complications.
